# NBS1 Heterozygosity and Cancer Risk

**DOI:** 10.2174/138920208784533610

**Published:** 2008-06

**Authors:** Alessandra di Masi, Antonio Antoccia

**Affiliations:** Department of Biology, University “Roma Tre”, Viale Guglielmo Marconi 446, I-00146 Roma, Italy

**Keywords:** NBS1, 657del5 mutation, R215W mutation, I171V mutation, IVS11+2insT mutation, heterozygous, cancer predisposition, lymphoma, breast cancer, prostate cancer, colorectal cancer.

## Abstract

Biallelic mutations in the *NBS1* gene are responsible for the Nijmegen breakage syndrome (NBS), a rare autosomal recessive disorder characterized by chromosome instability and hypersensitivity to ionising radiation (IR). Epidemiological data evidence that the *NBS1* gene can be considered a susceptibility factor for cancer development, as demonstrated by the fact that almost 40% of NBS patients have developed a malignancy before the age of 21. Interestingly, also *NBS1* heterozygotes, which are clinically asymptomatic, display an elevated risk to develop some types of malignant tumours, especially breast, prostate and colorectal cancers, lymphoblastic leukaemia, and non-Hodgkin’s lymphoma (NHL). So far, nine mutations in the *NBS1* gene have been found, at the heterozygous state, in cancer patients. Among them, the 657del5, the I171V and the R215W mutations are the most frequently described. The pathogenicity of these mutations is presumably connected with their occurrence in the highly conserved BRCT tandem domains of the NBS1 protein, which are present in a large superfamily of proteins, and are recognized as major mediators of processes related to cell-cycle checkpoint and DNA repair.

This review will focus on the current state-of-knowledge regarding the correlation between carriers of *NBS1* gene mutations and the proneness to the development of malignant tumours.

## INTRODUCTION

**Fig. (1):**
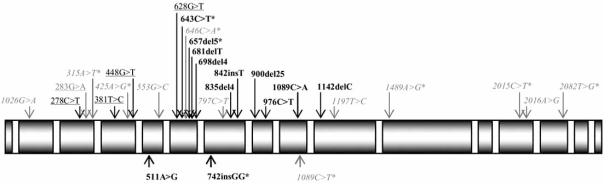
Coding sequence variants of *NBS1* gene identified to date (modified from http://www.nijmegenbreakagesyndrome.net). *Grey*: high frequency polymorphisms; grey*: low frequency polymorphisms; black: mutations found in NBS patients; black*: mutations also found in cancer patients at the heterozygous state; black: mutations found in cancer patients only; grey: mutation found both in cancer patients and in healthy controls.

Mutations at the homozygous status in the *NBS1* gene (known also as *NBN)* are responsible for a rare disease known as Nijmegen breakage syndrome (NBS; OMIM 251260), an autosomic recessive disorder whose signs are a distinct facial appearance, microcephaly, immunodeficiency, chromosome rearrangements and sensitivity to ionising radiation [1]. In NBS, a defective response to face DNA damage is associated with chromosomal instability, and in turn with a strong predisposition to develop malignancy, in particular lymphomas [2, 3]. The majority of them are non Hodgkin’s lymphoma (NHL) (with a high incidence of diffuse large B-cell lymphomas), lymphoblatic anaemia, and Hodgkin lymphoma (HL). Among solid tumours, medulloblastoma has been observed in four patients [4], and rhabdomiosarcoma of the perianal region in three others [5]. Since the latter is extremely uncommon among children, a strong association with NBS has been suggested [5]. All the 11 disease-causing mutations so far identified in *NBS1* gene have been found within exons 6-10 (Fig. **1**), and 8 of them result in premature truncation of the NBS1 protein, with the possible synthesis of NBS1 variants of lower molecular weight [6].

As a measure of cancer incidence in NBS, the Polish registry report that 40% of patients suffering for NBS developed lymphoma within the first two decades of life [3]. Though NBS is a recessive disease and one would not expect any cellular feature or clinical symptom, a growing number of papers report higher spontaneous and induced chromosome instability and an increased incidence of tumours among NBS carriers. The FISH chromosome painting analysis revealed that NBS carriers display a 3-fold higher rate of chromosome translocations compared with non-carriers [7]. Furthermore, the same authors reported that after irradiation, the response of cells from heterozygous carriers was intermediate between that of NBS homozygous and normal individuals, and could be clearly differentiated from those of the other groups in double-coded studies. Moreover, NBS heterozygosity can be distinguished from other genotypes by the number of the long-lived stable aberrations in NBS cells [8]. Radiation hypersensitivity in NBS carriers seems anyhow restricted to cells irradiated in the G_1_-phase, whereas the number of chromatid-aberrations scored in G_2_-phase-treated NBS heterozygous cells is in the range of normal cells or slightly higher [9].

Nine mutations localized in the coding sequence of the *NBS1 *gene have been found, at the heterozygous state, in cancer patients (Fig. **1**). The 657del5 (or founder mutation), the 511A>G (I171V), the 643C>T (R215W), and the 742insGG mutations were found both in NBS patients and in cancer patients. Four mutations have been found in cancer patients only (278C>T, 381C>T, 448G>T, and 628G>T). The 283G>A (D95N) mutation, has been identified both in cancer patients and in healthy controls [10], but is not listed as known *NBS1* polymorphism by the NCBI website [11]. 

## NBS1 MUTATIONS AND CANCER RISK

The first evidence of a possible correlation between NBS1 carriers and cancer risk, came from family data studies, indicating that blood relatives of NBS patients with the 657del5 founder mutation had a high probability to develop malignancy [12]. From 1998, several studies have examinated the frequency of the NBS1 mutations in cancer patients. In Table **1**, we have collected all the existing data relative to the frequency of cancer in heterozygotes for the commonest NBS1 gene mutations, in particular the 657del5, the R125W, the I171V, and the IVS11+2insT mutations. The relative distribution of these mutations in NBS1 heterozygous cancer patients is illustrated in Fig. (**2**).

### The 657del5 Mutation 

The 657del5 hypomorphic germ line mutation in exon 6 of *NBS1* gene, accounts for more than 90% of all mutant alleles in NBS. The highest frequency of heterozygous carriers of the 657del5 mutation has been found in the Slavic population of Central Europe, with an average frequency of 1/177 [13]. A total number of 81 carriers of 7474 cancer patients (1.8%), have been so far identified (Table **1**).

The evidence of a strong correlation between 657del5 mutation heterozygous carriers and cancer risk, has been strengthened by a large study on 344 blood relatives (first-through fourth-degree) of NBS patients, in 24 different NBS families of Czech Republic and Slovakia, from 1998 to 2003 [14]. Thirteen blood relatives developed malignancies of any type, among them eleven were carriers of the 657del5 *NBS1* mutation, compared with 6 expected. In this study, the most frequently type of cancer observed was stomach and colorectal cancer. Breast cancers were also reported, though at a lower frequency [14]. 

The cancer risk of the 657del5 mutation carriers has been also assessed in cancer patients with no NBS cases in the family. It was found that carriers of the 657del5 mutation were about twice more frequent among cancer patients than among matched controls [15]. Most of the 657del5 carriers were found among patients with melanoma (3.8%; OR: 6.376, p=0.0081), NHL (4.8%; OR: 8.05, p=0.0351), breast cancer (1.8%; OR: 2.927, p=0.0795), and colorectal cancer (1.3%; OR: 2.091, p=0.2197) [15]. Moreover, malignant tumours among parents and siblings of 657del5 carriers were twice more frequent (14/77) than in population control [15]. Interestingly, in a study on 2.400 healthy *NBS1* heterozygous Polish women, emerged a frequency of 96/10.603 (8.8%) malignant tumours among parents and siblings. This suggested that first-degree relatives of the 657del5 mutation carriers may have an elevated risk of cancer [15]. Heterozygotes for the 657del5 mutation are about three times more frequent among non-selected breast cancer patients than expected. Since an elevated risk of breast cancer has been also observed among carriers of mutations in the *BRCA1*, *p53* and *ATM* genes [16, 17], and because these gene products interact with each other and with NBS1 [18], these findings suggest that *NBS1* is another gene that might be associated with increased risk of breast cancer in heterozygotes [15]. 

### The R215W Mutation

The R215W mutation has been considered for a long time a polymorphism of *NBS1*, and only recently its severe pathogenicity is emerged with the identification of compound heterozygous 657del5/R215W NBS patients [19]. 

The R215W missense mutation was first described in a case of acute lymphoblastic leukemia (ALL) and in 9 probands of Slavic origin from a population-based study [10]. Subsequently, a high frequency of heterozygous carriers of the R215W mutation was found not only among children affected by ALL (2.1%), but also by HL (2.6%) (Table **1**) [20].

The R215W mutation has been also detected among several cancer patients, complexively in 34 of 4251 individuals with tumours (0.85%) (Table **1**). Several studies conducted among Poland, Germany, Czech Republic and United Kingdom, report that heterozygous carriers of the R215W missense mutation have an increased risk of colorectal cancer (1.3%), prostate cancer (1.6%), NHL (1.07%), and breast cancer (0.6%) (Table **1**) [15, 21-24]. 

### The I171V Mutation

The 511A>G (I171V) germ-line mutation was identified for the first time in 5 of 47 children with ALL [10]. These children were all characterized by late prognoses due to a late relapses [10]. The same mutation, at the homozygous state, was detected in a Japanese patient with aplastic anaemia, but with no other clinical signs of NBS [25]. In a large study aimed to assess the frequency of *NBS1* mutations in patients with larynx cancer and multiple primary tumours, is emerged that the frequency of the I171V mutation carriers is significantly higher than in population controls (2.3% in larynx cancer patients, p=0.0175; 5.4% in multiple primary tumours, p=0.0005). These results imply that the I171V mutation contributes significantly to the overall incidence of larynx carcinoma [26].

An investigation in the Polish population has provided evidences that the I171V mutation could be associated with an increased breast cancer risk (1.8% of I171V carriers, p=0.02) [27]. In particular, this association concerns patients with breast cancer, whose first-degree relatives also had diagnosis of those malignancies [27]. However, in an association study in two large hospital-based case-control settings from Germany and Belarus, is emerged that the I171V missense mutation does not significantly increase the breast cancer risk (0.9% of I171V carriers, p=0.7) [24].

Very recently, it has been shown that the 2.58% of studied patients with malignancies are carriers of the I171V mutation, compared to the 0.17% in the control group (p=0.0002). The percentage of the mutation carriers is particularly high among patients with neck and head tumours (6.17%, p=0.0001), thus suggesting that the I171V mutation in *NBS1* gene may be susceptibility factor in solid tumours [28]. 

### The IVS11+2insT Mutation

The IVS11+2insT mutation has been described for the first time in heterozygous Japanese subjects [29], with an increased risk of gastrointestinal cancer that originate in the stomach (2% of carriers, p<0.0001), and in the colorectum (0.8% of carriers, p=0.02) [29]. Interestingly, even if there is not a statistical significant correlation between carriers of the IVS11+2insT mutation and development of lung cancer, it is noteworthy that patients with this type of tumour and heterozygous for the IVS11+2insT mutation, are characterized by a relatively early onset [29]. Since it has been suggested the existence of a mendelian inheritance in the pathogenesis of lung cancer [30, 31], it has been proposed the possibility that the rare autosomal gene contributing to the early onset of lung cancer may be *NBS1* [29]. 

The IVS11+2insT mutation represent the first reported example of a germ line mutation that strongly predisposes to gastric cancer development in the Japanese population. Taking into account that each year 100.000 new case of gastric cancer arises in Japan [32], it is possible to hypothesize that heterozygous carriers of the IVS11+2insT mutation are susceptible to the development of gastric and colon carcinomas [29]. 

## BIOCHEMICAL EFFECTS OF THE NBS1 MUTATION ON THE STRUCTURE AND FUNCTION OF THE NBS1 PROTEIN

**Fig. (3):**
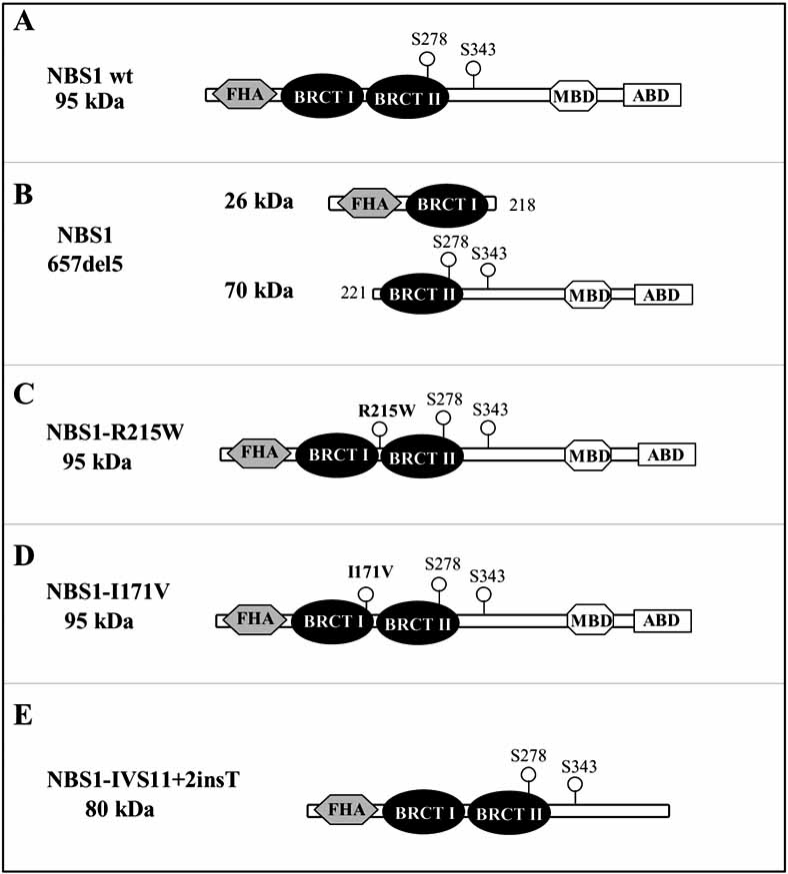
Wild type and mutated NBS1 proteins. (**A**) Structure of the NBS1 wild-type protein. (**B**) The 657del5 mutation, which splits up the tandem BRCT domains, determines the expression of two truncated proteins of 26 and 70kDa. (**C**) The R215W mutation occurs in the linker region that connects the two BRCT domains, and determines the substitution at position 215 of an arginine (R) with a tryptophan (W). (**D**) The I171V missense mutation occurs in the first BRCT domain, and determines the substitution at position 171 of an isoleucine (I) with a valine (V). (**E**) The NBS1 protein arising from the IVS11+2insT mutation, is characterised by the absence of the MRE11- and ATM-binding domain at the C-terminus. (MBD: MRE11 binding domain; ABD: ATM binding domain).

The NBS1 protein is part of a nuclear multi-protein complex composed also by MRE11 and RAD50 (MRN complex), which plays a crucial role in the response to DNA double strand breaks (DSBs), a lesion generated by both endogenous factors and environmental agents as ionising radiations [33, 34]. NBS1 consists of 754 amino acids, has a molecular weight of 95kDa, and is composed by three regions: the N-terminus, the central region, and the C-terminus (Fig. **3A**). The N-terminal region contains a fork-head associated (FHA) domain (amino acids 24-109) and two breast cancer C-terminus (BRCT) domain (BRCT1: amino acids 114-183; BRCT2: amino acids 221-291) [35-37]. The central region contains several consensus sequences for phosphorylation by ATM or ATR kinases [38-40]. The C-terminal region (amino acids 665-693) contains a MRE11 binding domain [41], and an ATM recruitment motif [42]. 

Low expression of abbreviated polypeptides of both N-terminal and C-terminal NBS1 has been demonstrated in NBS lymphoblastoid cell lines with different mutations. Particularly, C-terminal peptides of lower molecular weight than 95kDa, which maintain the ability to interact with MRE11, has been detected by means of a co-immunoprecipitation assay in lymphoblastoid NBS cell lines [6, 9]. NBS cells characterised by the presence of the classical mutation 657del5 in the *NBS1* gene, show two alternative forms of NBS1 with a lower molecular weight, of approximately 26 and 70 kDa. In particular, the 5bp deletion in position 657 splits the BRCT tandem domain exactly in the linker region that connects the two BRCT domains. The 26kDa protein includes the region 1-218 of the NBS1 protein, and comprises the FHA and the first BRCT domains. The 70kDa protein is produced by an alternative initiation of translation upstream the 5bp deletion: after a 18 residue extension at the N-terminus, the sequence is identical to that of the wild type NBS1, from the amino acid 221 to the end, and contains the second BRCT domain and the C-terminal half of NBS1 [37,43] (Fig. **3B**). 

The R215W missense mutation determines the substitution at position 215 of an arginine (R) with a tryptophan (W). The 215 residue of NBS1 protein is located at the C-terminus of the BRCT1 domain, right before the linker region which connects the two BRCT domains, and seems to be pivotal for the relative orientation of the NBS1 BRCT domains [44] (Fig. **3C**). Since tryptophan is a hydrophobic and bulky residue, it could lead to a perturbation of the relative geometry of the tandem BRCT domains. It has been demonstrated, in fact, that the R215W mutation in NBS1 impairs histone γ-H2AX binding after induction of DNA damage, leading to a delay in DNA-DSB rejoining [44]. 

The pathogenic character of the I171V mutation is presumably connected with its occurrence in the BRCT1 domain of NBS1 (Fig. **3D**). So, similarly to the R215W mutation, the I171V may perturb the proper geometry of the tandem BRCT domain, thus impairing the binding to γ-H2AX and the delocalization of the MRN complex to the vicinity of the DNA damage site [26,45].

The IVS11+2insT mutation determines the lacking of the MRE11- and ATM-binding domain at the C-terminus of the NBS1 protein [29] (Fig. **3E**). This determines the synthesis of an 80kDa protein, defective in the interaction with MRE11, MDC1, BRCA1 and ATM [29].

## CONCLUSIONS

Epidemiological data so far collected point to an increased risk of cancer incidence in heterozygous carriers of the 657del5, R215W, I171V, and IVS11+2insT mutations of the *NBS1* gene.

The only NBS1 657del5 founder mutation frequency among newborns is 1:154 in Czech Republic, 1:182 in Ukraine (Lvov region), 1:190 in Poland, with a mean prevalence of 1:177 for the three populations tested [13], whereas the NBS1 R215W mutation frequency among Czech newborns is 1:234 [46]. A moderately elevated risk in heterozygous carriers would results in hundreds of new cancer cases in these populations every year. Because it cannot be excluded that cancer patients who carry germ-line *NBS1* mutations may show a specific sensitivity to treatment with ionising radiation or cytostatic drugs, as recently shown [8], systematic studies are now under way to protocol their responses to radio- and chemotherapy.

Experimental support that *NBS1 *heterozygosity predispose cells to malignancy, come from a study in which the mouse homologue of the human *NBS1* gene, *Nbn*, was disrupted in mice [47]. *Nbn*^+/-^ mice showed a significantly increased occurrence of spontaneous solid tumours (epithelial tumours affecting the liver, prostate and mammary glands, and gonad malignancy) in addition to lymphoma. Moreover, ionising radiation dramatically increased cancer formation in *Nbn*^+/-^ mice, especially thyroid tumours. These data provide a clear relationship between *NBS1* heterozygosity, radiation sensitivity and increased cancer risk. Interestingly, examination of the tumours gave no evidence for loss or mutation of the wild-type allele, suggesting that haploinsufficiency is the presumed pathogenic mechanism. In contrast, for human heterozygotes, the possible existence of a truncated protein produced by alternative translation [6], and capable of interaction with MRE11 would be compatible with a dominant negative mechanism.

## Figures and Tables

**Fig. (2):**
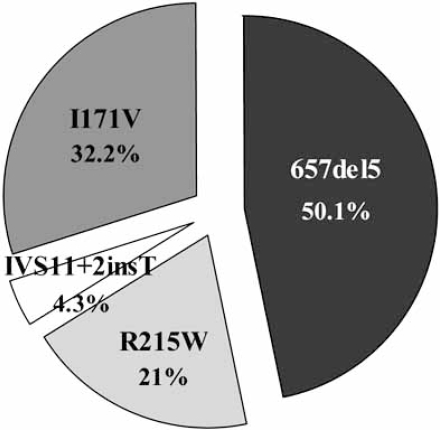
Distribution of the 657del5, R215W, I171V, and IVS11+ 2insT NBS1 gene mutations-type among *NBS1* heterozygotes affected with tumour.

**Table 1:** Frequency of the 657del5, R215W, I171V, and IVS11+2insT NBS1 Gene Mutations-Type Among NBS1 Heterozygotes Tumour and Control Subjects (NHL: Non-Hodgkin Lymphoma; ALL: Acute Lymphoblastic Leukaemia; HL: Hodgkin Lymphoma). (nad: Not Available Data)

*NBS1* Mutation	Patient Age	Cancer Type	N° of *NBS1* Mutation Carriers Among Tumour Patients	N° of *NBS1* Mutation Carriers Among Control Subjects	Statistical Analysis	Ref.
657del5	adult	colorectal	3/234 (1.3%)	10/1620 (0.6%)	OR: 2.091, p=0.2197	[15]
		breast	4/224 (1.8%)	10/1620 (0.6%)	OR: 2.927, p=0.0795	[15]
			11/562 (1.96%)	nad	OR: 3.21, p = 0.0107	[48]
			1/477 (0.2%)	1/866 (0.1%)	OR: 1.8, p=0.76	[49]
			7/873 (0.8%)	2/692 (0.3%)	OR: 2.8, p=0.32	[50]
			2/2012 (0.8%)	18/4000 (0.5%)	OR: 1.9, p=0.09	[51]
			2/150 (1.3%)	3/530 (0.56%)	χ^2^test, p<0.0001	[51]
			3/80 (3.75%)	3/530 (0.56%)	χ^2^ test, p<0.0001	[51]
		melanoma	4/105 (3.8%)	10/1620 (0.6%)	OR: 6.376, p=0.0081	[15]
			1/376 (0.26%)	0	nad	[23]
		NHL	2/42 (4.8%)	10/1620 (0.6%)	OR: 8.05, p=0.0351	[15]
			8/228 (3.5%)	10/1620 (0.6%)	OR: 5.85, p=0.0001	[22]
		prostate	5/56 (9%) (familial)	9/1500 (0.6%)	OR: 16, p>0.0001	[52]
			7/305 (2.2%)(non familial)	9/1500 (0.6%)	OR: 3.9, p=0.01	[52]
		gastrointestinal lymphoma	4/37 (10.8%)	10/1620 (0.6%)	OR: 19.52, p=0.0002	[22]
	children	NHL	1/68 (1.5%)	11/2261 (0.49%)	χ^2^ test, p<0.0001	[53]
			2/212 (0.9%)	42/6984 (0.6%)	OR: 1.57, p=0.041	[54]
		ALL	3/270 (1.1%)	42/6984 (0.6%)	OR: 1.85, p=0.035	[54]
			1/68 (1.5%)	11/2261 (0.49%)	χ^2^test, p<0.0001	[53]
		ALL+NHL+HL	5/545 (0.9%)	42/6984 (0.6%)	OR: 1.48, p=0.037	[54]
		ALL+NHL	5/482 (1.03%)	42/6984 (0.6%)	OR: 1.73, p=0.029	[54]
			2/68 (2.9%)	0	nad	[55]
R215W	adult	colorectal	3/234 (1.3%)	4/1620 (0.2%)	OR: 5.247, p=0.0472	[15]
		breast	9/1588 (0.6%)	5/1014 (0.5%)	OR: 1.9, p=0.18	[24]
			9/1076 (0.8%)	2/1017 (0.2%)	OR: 1.9, p=0.18	[24]
		melanoma	1/376 (0.26%)	nad	nad	[23]
		NHL	2/186 (1.07%)	10/1620 (0.6%)	nad	[22]
		prostate	6/338 (1,78%) (sporadic)	3/208 (1.4%)	OR: 1.24, p =0.77	[21]
			2/139 (1,44%) (non familial)	3/208 (1.4%)	OR: 1, p=1	[21]
	children	HL	1/39 (2.6%)	nad	nad	[20]
		ALL	1/47 (2.1%)	nad	nad	[10]
I171V	adult	larynx cancer	4/176 (2.3%)	1/500 (0.2%)	OR: 11.7, p=0.0175	[26]
		multiple primary tumors	5/93 (5.4%)	1/500 (0.2%)	OR: 28.35, p=0.0005	[26]
		breast	20/1636 (1.2%)	18/1014 (1.8%)	OR: 0.68, p=0.3	[24]
			10/1048 (0.9%)	7/1017 (0.7%)	OR: 1.39, p=0.7	[24]
			5/270 (1.8%)	1/500 (0.2%)	OR: 9.42, p=0.02	[27]
		head and neck	5/81 (6.17%)	1/600 (0.17%)	OR: 39.41, p=0.0001	[28]
		colorectal carcinoma	3/131(2.29%)	1/600 (0.17%)	OR: 14.39, p=0,0196	[28]
IVS11+2insT	adult	lung	2/532 (0.4%)	2/2348 (0.08%)	OR: 4.43, p=0.16	[29]
		gastric cancer	2/472 (0.4%)	2/2348 (0.08%)	OR: 25, p<0.0001	[29]
		colorectal cancer	3/472 (0.6%)	2/2348 (0.08%)	OR: 9.43, p=0.02	[29]

## ABBREVIATIONS

NBS1: = Nijmegen breakage syndrome 1 mutated gene
NBS: = Nijmegen breakage syndrome
IR: = Ionising radiation
BRCT: = BRCA1 C-terminal domain
NHL: = Non Hodgkin lymphoma
HL: = Hodgkin lymphoma
FISH: = Fluorescence in situ hybridization
OR: = Odds ratio
ATM: = Ataxia telangiectasia mutated gene
ALL: = Acute lymphoblastic leukaemia
MRN: = MRE11/RAD50/NBS1 complex
DSBs: = Double strand breaks
FHA: = Forkhead associated domain
ATR: = Ataxia telangiectasia related
MDC1: = Mediator of DNA damage checkpoint
